# A one-step reverse transcription loop-mediated isothermal amplification for detection and discrimination of infectious bursal disease virus

**DOI:** 10.1186/1743-422X-8-108

**Published:** 2011-03-08

**Authors:** Yongqiang Wang, Zhonghui Kang, Honglei Gao, Yulong Gao, Liting Qin, Huan Lin, Fei Yu, Xiaole Qi, Xiaomei Wang

**Affiliations:** 1 Division of Avian Infectious Diseases, State Key Laboratory of Veterinary Biotechnology, Harbin Veterinary Research Institute, the Chinese Academy of Agricultural Sciences, Harbin, Heilongjiang 150001, China

## Abstract

**Background:**

Infectious bursal disease (IBD) is a highly contagious immunosuppressive disease in young chickens caused by infectious bursal disease virus (IBDV). It causes huge economic losses to the poultry industry. The objective of this study is to develop a loop-mediated isothermal amplification (LAMP) method for the detection and discrimination of IBDV.

**Results:**

In this study, we applied reverse transcription loop-mediated isothermal amplification (RT-LAMP) to detect IBDV in one simple step and further identified the very virulent strain from non-vvIBDVs with a simply post-amplification restriction enzyme analysis. Based on sequence analysis, a set of two inner, two outer and two loop primers were designed to target the VP5 gene and they showed great specificity with no cross reaction to the other common avian pathogens. The detection limit determined by both color change inspection and agarose gel electrophoresis was 28 copies viral RNA, which was almost as sensitive as a real-time RT-PCR previous developed in our laboratory. We also identified a unique Tfi I restriction site located exclusively in non-vvIBDVs, so very virulent strain could be distinguished from current vaccine strains. By screening a panel of clinical specimens, results showed that this method is high feasible in clinical settings, and it obtained results 100% correlated with real-time RT-PCR.

**Conclusion:**

RT-LAMP is a rapid, simple and sensitive assay. In combination with the Tfi I restriction analysis, this method holds great promises not only in laboratory detection and discrimination of IBDV but also in large scale field and clinical studies.

## Background

Infectious bursal disease virus (IBDV) is the etiologic agent of infectious bursal disease (IBD), an acute and highly contagious disease affecting young chickens. Characterized by immunosuppression and a high rate of mortality, this disease causes a huge economic loss to the poultry industry worldwide [1]. In recent years, IBD has rarely showed the typical clinical symptoms and become less responsive to the conventional vaccination. Very virulent IBDV (vvIBDV) causing severe mortality in chickens has become the dominant strain responsible for several disease outbreaks in China [2]. To control this disease, a sensitive, reliable, rapid and clinically feasible method for the detection of the virus and identification of the very virulent strain at early stage of infection is urgently needed.

Developed by Notomi et al., loop-mediated isothermal amplification (LAMP) is a novel DNA amplification method with high specificity and sensitivity under isothermal condition [3]. It is also a robust method that produces a high amount of products sufficient for real time monitoring by visual inspection. In addition, RNA can be directly used as starting material by reverse transcription coupled with loop-mediated isothermal amplification (RT-LAMP) in one step [4-8], making it ideal for detection of RNA-viruses such as IBDV. Previously, in a field diagnostic testing, RT-LAMP showed great superiority over conventional RT-PCR [9,10]. More recently, it has been successfully applied for the detection of IBDV [11,12]. However, none of these studies differentiated virus types.

In this study, we applied RT-LAMP to detect IBDV in one simple step and further identified the very virulent strain from the non-vvIBDVs with a post-amplification restriction digestion analysis. We show here that this method is very efficient and convenient compared with conventional RT-PCR and real-time RT-PCR, and also high feasible with clinical specimens.

## Methods

### Virus strains

IBDV Gt strain was attenuated from the vvIBDV Gx strain through continuous passage in specific-pathogen-free chicken embryos for 5 generations and in chicken embryo fibroblasts for 20 generations [13]. IBDV Gt, IBDV D78, vvIBDV Gx and chicken anemia virus (CAV) M9905 were all stock strains of our laboratory. Other avian pathogens, such as avian influenza virus (AIV) A/Chicken/Shandong/6/96 (H9N2), Newcastle disease virus (NDV) La sota, infectious bronchitis virus (IBV) F and Marek's disease virus (MDV) CV1988 were obtained from the Harbin Veterinary Research Institute, China.

### Sequence analysis and primer design

**Figure 1:**
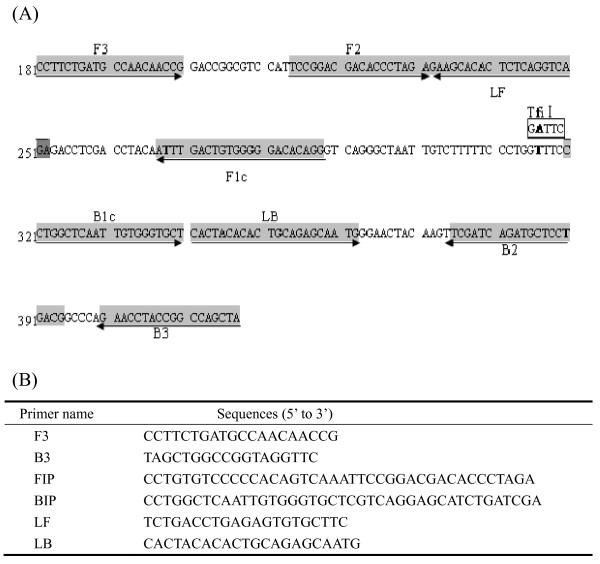
**Primer design for RT-LAMP to detect IBDV based on the VP5 gene of Gx strain**. (A). Genomic DNA sequence of vvIBDV Gx strain (GenBank Accession number: [AY444873]) VP5 gene from 181 to 417 nt in orientation 5' to 3'. Nucleotide sequences used for the primers are highlighted with grey background, and the arrows indicate the 5' to 3' direction of the primers. The open box shows the Tfi I restriction site exclusively located in non-vvIBDVs. (B). A table listing the names and sequences of all 6 primers.

Sequence data for 57 IBDV isolates including vvIBDVs (GenBank accession numbers: [AF092943], [AF240686], [AF247006], [AF262030], [AF322444], [AF362776], [AF508176], [AF527039], [AF533670], [AJ318896], [AJ879932], [AY099456], [AY134874], [AY323952], [AY444873], [AY520909], [AY520910], [AY520911], [AY598356], [AY665672], [AY769978], [AY780418], [D49706], [DQ286035], [DQ927042], [EF517528]) and non-vvIBDVs (GenBank accession numbers: [AF006694], [AF051837], [AF109154], [AF133904], [AF194428], [AF321054], [AF321055], [AF362747], [AF362771], [AF362773], [AF499929], [AJ310185], [AY029166], [AY319768], [AY368653], [AY462026], [AY918948], [AY918950], [D00499], [D00867], [D00868], [D00869], [DQ187988], [DQ403248], [EF418033], [EF418034], [EF418035], [M66722], [X03993], [X16107], [X84034]) were retrieved from GenBank, and analyzed with the sequence analysis software MegAlign (DNAStar Inc., Madison, WI, USA). Sequence alignment was performed using the Clustal W multiple sequence alignment program. The sequence encoding the VP5 protein was chosen as the target sequence for RT-LAMP. Six primers specific for the VP5 gene including two outer primers (F3 and B3), two inner primers (FIP and BIP) and two loop primers (LF and LB) were designed with the Primer Explorer V4 software (https://primerexplorer.jp) (Figure 1).

### RNA extraction

Viral RNA was extracted using TRIzol Reagent (Invitrogen, USA) according to manufacturer's instructions. RNA was dissolved in 20 μL DEPC-treated water, and stored at -70°C before use.

### RT-LAMP reaction

The RT-LAMP reaction was carried out using a Loopamp RNA amplification kit (Eiken Chemical Co., Ltd, Tokyo, Japan). Each 25 μL reaction contained 1.6 μM of each inner primer (FIP and BIP), 0.8 μM of each loop primer (LF and LB), 0.2 μM of each outer primer (F3 and B3), and 2 μL template RNA. In the reaction, 1 μL of fluorescent reagent FDR (Eiken Chemical Co., Ltd, Tokyo, Japan) was added to detect amplified products. After initial optimization of reaction conditions under different temperatures (61-65°C) for various times (15-60 min), a 65°C incubation for 60 minutes yielded the best result (not shown), therefore, all the LAMP reactions in the study presented in "Results" were carried out at 65°C for 1 hour, and inactivated at 80°C for 10 min.

The RT-LAMP product was analyzed by agarose gel electrophoresis and also visually inspected for the color change from orange color to bright green. For electrophoresis, 10 μL aliquot of RT-LAMP product was separated on a 2% agarose gel, stained with ethidium bromide, and photographed under a UV transilluminator. For the visual inspection, the tubes were observed by naked eyes and photographed under the natural light. The color of a negative control reaction should have remained orange.

### Specificity test

To evaluate the cross-reactivity of the VP5 primer set with other common avian pathogens, samples extracted from AIV, NDV, IBV, MDV and CAV strains were tested together with vvIBDV Gx strain and a DEPC-treated water negative control under the same conditions

### Sensitivity test

To evaluate the sensitivity of RT-LAMP, RNA standards were in vitro transcribed with T7 Cap-Scribe (Roche, Germany) from plasmid pcDNA3.1-GtVP5 carrying the VP5 gene of Gt strain under the control of T7 promoter. RNA was quantified by spectrophotometer, and then 10-fold serially diluted from 2.8 × 10^5 ^copies/μL to 2.8 × 10^0 ^copies/μL and used as templates for RT-LAMP. The lowest amount of RNA detectable under the conditions described above was defined as the detection limit.

### Identification of vvIBDV by Tfi I restriction fragment analysis

RT-LAMP products were digested with Tfi I in a 20 μL reaction containing 3 μL RT-LAMP product, 1 × NEBuffer 3, 1 × BSA and 2.5 U TfiI (New England Biolabs, USA). After incubation at 65°C for 1 hour, 10 μL aliquot was subjected to electrophoresis on 2% agarose gel and stained with ethidium bromide. The DNA band pattern was visualized with a UV transilluminator and photographed.

### Clinical specimen evaluation

From 1999 to 2008, samples from Bursa of Fabricius in chickens exhibiting skeptical pathologic features of IBD were collected in different commercial broiler and layer farms from 11 provinces of China. All specimens were processed according to the International Cooperation with Developing Countries project method. Tissues were homogenized as described previously [2]. Viral RNA extraction, RT-LAMP, product analysis and Tfi I digestion were carried out essentially the same as described above.

#### Reverse transcription

12 μL viral RNA and the segment specific primer R (5'-CCATTGTAGCTAACATCTGTC-3') were denatured at 95°C for 5 min and chilled immediately on ice for 2 min. Reverse transcription was performed in a 20 μL containing 12 μL RNA, 4 μL of 5 × FS buffer (Invitrogen, USA), 1 μL of dNTP (10 mM each), 1 μL of DTT (0.1 M), 1 μL of specific primer R (5'-CCATTGTAGCTAACATCTGTC-3' 50 μM), 100 U of Superscript™ III; Reverse Transcriptase (Invitrogen, USA), 20 U of RNase Inhibitor (TaKaRa, China). Reaction was carried out at 50°C for 1 h and 70°C for 15 min. 2 μL cDNA was used in conventional PCR and real-time PCR reactions below.

#### PCR

using a pair of primers (F: 5'-GCGAATTCGGATACGATCGGTCTG-3'; R: 5'-CCATTGTAGCTAACATCTGTC-3') and Ex Taq polymerase (TaKaRa, China), a conventional PCR was carried out with a pre-denaturation at 95°C for 5 min and 30 cycles of 94°C for 30 sec, 50°C 30 sec, 72°C for 45 sec, followed by 72°C for 7 min. PCR product was electrophoresed on 1% agarose gel and stained with ethidium bromide. The correct amplification product showed as a DNA band of bout 560 bp.

#### Real-time PCR

the TaqMan based real-time PCR was performed in a total volume of 25 μL as described in our previous paper [14], and the reaction was performed with a pre-denaturation at 95°C for 5 min, and 40 cycles of denaturation at 95°C for 10 sec and annealing/elongation at 60°C for 40 sec. Fluorescent signal measurements were carried out during the elongation step.

## Results

### Specificity and Sensitivity of the RT-LAMP

**Figure 2:**
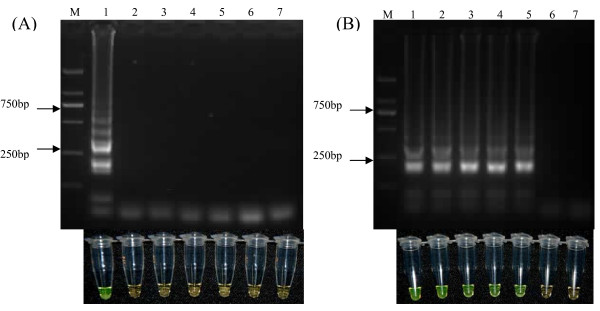
**Specificity and sensitivity of RT-LAMP for the detection of IBDV**. (A) Five related avian pathogens and vvIBDV Gx strain were subjected to RT-LAMP using the primers shown in Figure 1, and the RT-LAMP products were examined by both agarose gel electrophoresis (upper panel) and visually inspection for color changes (lower panel). Lanes M, DNA marker DL2000 (TaKaRa, China, with bands of 2000, 1000, 750, 500, 250 and 100 bp); 1, vvIBDV Gx strain; 2, avian influenza virus A/Chicken/Shandong/6/96 (H9N2) strain; 3, Newcastle disease virus La sota strain; 4, infectious bronchitis virus F strain; 5, Marek's disease virus CV1988 strain; 6, chicken anemia virus M9905 strain; 7, DEPC-treated water. (B) RNA standards in vitro transcribed and serially diluted were subjected to RT-LAMP and the RT-LAMP products were examined by both agarose gel electrophoresis (upper panel) and visually inspection for color changes (lower panel). Lanes M, DNA marker DL2000 (TaKaRa, China); 1-6, 2.8 × 10^5^, 2.8 × 10^4^, 2.8 × 10^3^, 2.8 × 10^2^, 2.8 × 10^1 ^and 2.8 × 10° copies of RNA, respectively; 7, DEPC-treated water.

As shown in Figure 2A, RT-LAMP products of RNA from vvIBDV Gx showed a ladder-like pattern on the gel. The reaction also caused change of turbidity, the color inside the tube changed from orange to green that was easily visible to naked eyes under the natural light. The reactions containing samples of common avian pathogens AIV, NDV, IBV, MDV and CAV as well as the negative control showed no product on the gel. Consistently, the color of these negative reactions remained orange.

Upon 10-fold serial dilution, RNA standards with known copy numbers (2.8 × 10^5 ^copies/μL to 2.8 × 10° copies/μL) were used for RT-LAMP. As shown in Figure 2B, RT-LAMP successfully detected as little as 28 copies of RNA molecules, determined by both the agarose gel electrophoresis and color change inspection.

From the above two sets of experiments, a good correlation between results from the gel images and that from the color change was observed.

### Tfi I mediated vvIBDV discrimination

**Figure 3:**
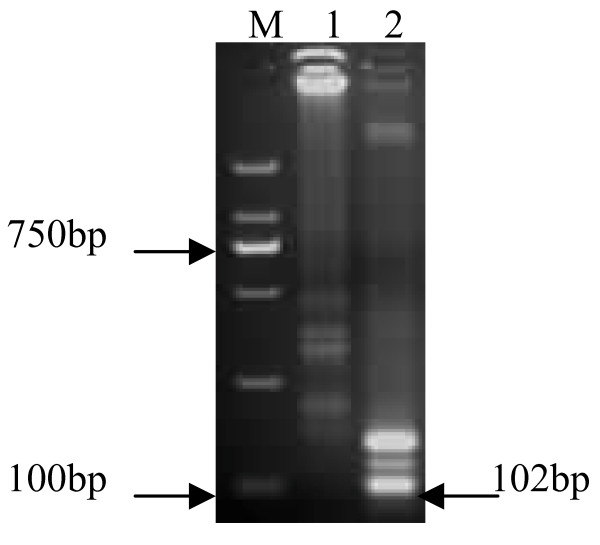
**Different Tfi I restriction patterns of RT-LAMP products of vvIBDV Gx and attenuated Gt strains**. RT-LAMP products were digested by Tfi I and subjected to a 2% agarose gel. After ethidium bromide staining, DNA band patterns were photographed under a UV transilluminator. Lanes M, DNA marker DL2000 (TaKaRa, China, with 2000, 1000, 750, 500, 250 and 100 bp bands); 1, RT-LAMP product of Gx digested by Tfi I; 2, RT-LAMP product of Gt digested by Tfi I.

Sequence analysis based on 26 isolates of vvIBDV and 31 isolates of non-vvIBDV revealed a unique Tfi I restriction site (^5'^GAWTC^3' ^W = A or T) located between the F1c and B1c regions, and significantly, this site exists exclusively in non-vvIBDVs including typical classical, variant and attenuated strains, therefore, this Tfi I restriction site determined by a single nucleotide polymorphism (SNP) can discriminate vvIBDVs from non-vvIBDVs (Table 1 and Table 2). After digestion of RT-LAMP products with the Tfi I, as expected, vvIBDV and non-vvIBDV showed different restriction patterns on agarose gel. After 1 hour digestion, a new 102 bp fragment was observed in sample from Gt strain but not in that of Gx (Figure 3). After digestion for as long as 15 hours, sample from vvIBDV Gx retained its ladder-like DNA band pattern on the gel (not shown).

**Table 1:** Nucleotide sequences of vvIBDVs at the TfiI site.

Strains/isolates	Accession number	Geographic origin	Nucleotides at the restriction site
Gx	AY444873	China	G T T T C^Note^
Harbin-1	EF517528	China	. . . . .
SH95	AY134874	China	. . . . .
GZ/96	AY598356	China	. . . . .
HK46	AF092943	Hongkong	. . . . .
Chinju	AF508176	Korea	. . . . .
SH/92	AF533670	Korea	. . . . .
OKYM	D49706	Japan	. . . . .
SDH1	AY323952	Iran	. . . . .
BD 3/99	AF362776	Bangladesh	. . . . .
UPM97/61	AF247006	Malaysia	. . . . .
UPM94/273	AF527039	Malaysia	. . . . .
UPM92-04	AF262030	Malaysia	. . . . .
B00/73	AY520909	Malaysia	. . . . .
B00/81	AY520910	Malaysia	. . . . .
94230	AY520911	Malaysia	. . . . .
Tasik94	AF322444	Indonesia	. . . . .
T09	AY099456	Nigeria	. . . . .
PO7	AY665672	Tunisia	. . . . .
ks	DQ927042	Israel	. . . . .
UK661	AJ318896	U.K.	. C . . .
02015.1	AJ879932	France	. . . . .
D6948	AF240686	Netherlands	. . . . .
Ipumirim-BR	AY769978	Brazil	. . . . .
SM-BR	AY780418	Brazil	. . . . .
MG7	DQ286035	Brazil	. . . . .

^Note^The TfiI site was ^5'^GAWTC^3' ^W = A or T. Dark dot indicated residue which was identical to the vvIBDV Gx. There was no TfiI site in vvIBDVs.

**Table 2:** Nucleotide sequences of non-vvIBDVs at the TfiI stie.

Strains/isolates	Accession number	Geographic origin	Phenotype	Nucleotides in the restriction site
Gt	DQ403248	China	Attenuated	G A T T C^Note^
HZ2	AF321054	China	Attenuated	. . . . .
JD1	AF321055	China	Attenuated	. . . . .
CJ801bkf	AF006694	China	Attenuated	. . . . .
GZ29112	AF051837	China	Attenuated	. . . . .
NB	AY319768	China	Attenuated	. . . . .
CEF94	AF194428	Netherlands	Attenuated	. . . . .
D78	AF499929	Luxembourg	Attenuated	. . . . .
CT	AJ310185	France	Attenuated	. . . . .
Cu-1 M	AF362771	Germany	Attenuated	. . . . .
P2	X84034	Germany	Attenuated	. . . . .
Edgar T	AY462026	USA	Attenuated	. . . . .
002-73	X03993	Australia	Classical	. . . . .
CU-1	X16107	Germany	Classical	. . . . .
CS-2-35	EF418033	USA	Classical	. . . . .
GA-1	EF418034	USA	Classical	. . . . .
H-30	EF418035	USA	Classical	. . . . .
P3009	AF109154	Taiwan	Classical	. . . . .
A-BH83	DQ187988	Brazil	Classical	. . . . .
STC	D00499	USA	Classical	. . . . .
Cu1	D00867	Germany	Classical	. . . . .
PBG-98	D00868	U. K.	Classical	. . . . .
52/70	D00869	U. K.	Classical	. . . . .
IM	AY029166	USA	Classical	. . . . .
Cu-1 wt	AF362747	Germany	Classical	. . . . .
Lukert	AY918948	USA	Classical	. . . . .
Edgar C	AY918950	USA	Classical	. . . . .
GLS	AY368653	USA	Variant	. . . . .
varient E	AF133904	USA	Variant	. . . . .
23/82	AF362773	U. K.	Serotype II;	. . . . .
OH	M66722	Canada	Serotype II;	. . . . .

^Note^The TfiI site was ^5'^GAWTC^3' ^W = A or T. Dark dot indicated residue which was identical to the attenuated Gt. There was a TfiI site in non-vvIBDVs.

### Evaluation of RT-LAMP for clinical specimens

To evaluate the feasibility of RT-LAMP for detecting IBDV in clinical specimens, 48 clinical specimens were obtained from a wide range of geographic locations and assayed by RT-LAMP. In parallel, conventional RT-PCR and real-time RT-PCR were also performed. As summarized in Table 3, percentage of positive samples detected by conventional RT-PCR, real-time RT-PCR and RT-LAMP were 79.2%, 95.8% and 95.8%, respectively. The results of RT-LAMP and real-time RT-PCR were 100% correlated and the correlation between RT-LAMP and conventional RT-PCR was 83.3%.

**Table 3:** Detection rates of clinical specimens by RT-PCR, real-time RT-PCR and RT-LAMP

Source (NO. of specimens)	RT-PCR		real-time RT-PCR		RT-LAMP	
	
	Positive	Negative	Positive	Negative	Positive	Negative
Hei Longjiang (18)	17	1	18	0	18	0
Shan Dong (9)	6	3	8	1	9	0
Ji Lin (6)	6	0	6	0	6	0
Jiang Su (2)	1	1	2	0	1	1
Jiang Xi (1)	0	1	1	0	1	0
Shang Hai (1)	1	0	1	0	1	0
Guang Xi (1)	1	0	1	0	1	0
Yun Nan (1)	1	0	1	0	1	0
Hu Nan (1)	1	0	1	0	1	0
Hu Bei (1)	1	0	1	0	1	0
Fu Jian (1)	1	0	1	0	1	0
-^a ^(6)	2	4	5	1	5	1
Total (48)	38	10	46	2	46	2
Percent (%)	79.2	20.8	95.8	4.2	95.8	4.2

^a^indicates unknown background information on the specimens.

The RT-LAMP products of the 46 positive specimens were subsequently digested by Tfi I together with those from the vvIBDV control Gx and non-vvIBDV control D78. Among those, only one clinical specimen showed a 102 bp fragment as well as the negative control from non-vvIBDV D78 (not shown), indicating that 97.8% (45/46) of the infected specimens or 93.8% (45/48) of total specimens were infected by vvIBDV.

## Discussion

In this study, we developed a RT-LAMP assay for the detection of IBDV and subsequent discrimination of vvIBDV based on a SNP site in its VP5 gene. The use of loop primers in this assay greatly accelerates the reaction [15-20]. We show here that the primers did no cross react with a panel of other common avian pathogens, and the assay had a high sensitivity with the detection limit of 28 copies, which is almost as sensitive as a real-time RT-PCR-based assay for the same virus we developed in the earlier study [14] and 100 times greater than the conventional RT-PCR [17,20-22]. RT-LAMP is more sensitive than the conventional RT-PCR and more convenient than real-time RT-PCR. Another advantage of this assay is that the results can be examined by inspection of color change and examination with agarose gel electrophoresis. Consistence results observed by both methods in this study indicate that a visual inspection is sufficient for a routine test [23]. This is particular useful and can be extremely convenient in a large scale screening process.

In clinical specimens, 93.8% was positive for vvIBDV infection, indicating the severity of vvIBDV infection in many areas of China. Even though vaccination has been widely adopted, vvIBDV can break through high levels of maternal antibodies in commercial flocks [24,25]. Since vaccine was produced by attenuated or classical strains, it is very important and significant that wild isolates of vvIBDV can be distinguished from vaccinated strains.

Sequence analysis showed a SNP in the target sequence of RT-LAMP among IBDV strains. "A" is conserved in classical, attenuated, variant and serotype II strains, creating a Tfi I site in this site, while it is substituted by "T" in typical vvIBDV strains except UK661 that has a "C". Since this SNP was identified from strains with a wide geographic distribution, so the Tfi I digestion based on this SNP should be reliable and generally work. Although the VP5 of infectious bursal disease virus has been reported to contribute to rival virulence and viral release [26,27], the role of this nucleotide substitution in viral pathogenesis is still unknown. We are yet to determine whether this point mutant may be involved in the virulence or viral release, or it may just be a unique nucleotide tag between vvIBDV and non-vvIBDVs.

## Conclusion

In summary, one-step RT-LAMP is a rapid, efficient, sensitive and highly specific assay for the identification of IBDV. In combination with Tfi I restriction analysis, vvIBDV strain can be discriminated from non-vvIBDVs. Owning to these properties, this method showed great promises not only in laboratory test but also in the field and clinical applications.

## Competing interests

The authors declare that they have no competing interests.

## Authors' contributions

YQW and XMW designed this study; YQW wrote the paper; YQW and ZHK carried out this study; YQW, ZHK, HLG and XMW analyzed the data; HLG, YLG, LTQ, HL and XLQ collected the clinical samples; YQW, YLG and FY revised the manuscript critically. All of the authors read and approved the final version of this manuscript.

## References

[B1] Cosgrove AS An apparently new disease of chicken-avian nephrosis Avian Dis 1962 6 385 389 10.2307/1587909

[B2] Yuwen YQ Gao YL Gao HL Qi XL Li TQ Liu W Wang XM Sequence analysis of the VP2 hypervariable region of eight very virulent infectious bursal disease virus isolates from the northeast of China Avian Dis 2008 52 284 290 10.1637/8175-111707-Reg.1 18646458

[B3] Notomi T Okayama H Masubuchi H Yonekawa T Watanabe K Amino N Hase T Loop-mediated isothermal amplification of DNA Nucleic Acids Res 2000 28 e63 10.1093/nar/28.12.e63 10871386 PMC102748

[B4] Chen HT Zhang J Sun DH Ma LN Liu XT Cai XP Liu YS Development of reverse transcription loop-mediated isothermal amplification for rapid detection of H9 avian influenza virus J Virol Methods 2008 151 200 203 10.1016/j.jviromet.2008.05.009 18572258

[B5] Kiatpathomchai W Jareonram W Jitrapakdee S Flegel TW Rapid and sensitive detection of taura syndrome virus by reverse transcription loop-mediated isothermal amplification J Virol Methods 2007 146 125 128 10.1016/j.jviromet.2007.06.007 17643501

[B6] Le Roux CA Kubo T Grobbelaar AA Jansen van Vuren P Weyer J Nel LH Swanepoel R Morita K Paweska JT Development and evaluation of a real-time reverse transcription-loop-mediated isothermal amplification assay for rapid detection of rift valley fever virus in clinical specimens J Clin Microbiol 2009 47 645 651 10.1128/JCM.01412-08 19109471 PMC2650915

[B7] Li Q Zhou QF Xue CY Ma JY Zhu DZ Cao YC Rapid detection of porcine reproductive and respiratory syndrome virus by reverse transcription loop-mediated isothermal amplification assay J Virol Methods 2009 155 55 60 10.1016/j.jviromet.2008.09.012 18926852

[B8] Peyrefitte CN Boubis L Coudrier D Bouloy M Grandadam M Tolou HJ Plumet S Real-time reverse-transcription loop-mediated isothermal amplification for rapid detection of rift valley fever virus J Clin Microbiol 2008 46 3653 3659 10.1128/JCM.01188-08 18799705 PMC2576582

[B9] Dukes JP King DP Alexandersen S Novel reverse transcription loop-mediated isothermal amplification for rapid detection of foot-and-mouth disease virus Arch Virol 2006 151 1093 1106 10.1007/s00705-005-0708-5 16453084

[B10] Parida MM Santhosh SR Dash PK Tripathi NK Saxena P Ambuj S Sahni AK Lakshmana Rao PV Morita K Development and evaluation of reverse transcription-loop-mediated isothermal amplification assay for rapid and real-time detection of Japanese encephalitis virus J Clin Microbiol 2006 44 4172 4178 10.1128/JCM.01487-06 17005741 PMC1698363

[B11] Xu JT Zhang ZM Yin YB Cui SJ Xu SZ Guo YY Li JD Wang JL Liu XC Han LM Development of reverse-transcription loop-mediated isothermal amplification for the detection of infectious bursal disease virus J Virol Methods 2009 162 267 271 10.1016/j.jviromet.2009.07.010 19643144

[B12] Xue CY Zhang Y Zhou QF Xu C Li XM Cao YC Rapid detection of Infectious bursal disease virus by reverse transcription loop-mediated isothermal amplification assay J Vet Diagn Invest 2009 21 841 843 19901286 10.1177/104063870902100612

[B13] Wang XM Zeng XW Gao HL Fu CY Wei P Changes in VP2 gene during the attenuation of very virulent infectious bursal disease virus strain Gx isolated in China Avian Dis 2004 48 77 83 10.1637/7061 15077800

[B14] Wang YQ Qi XL Gao HL Gao YL Lin H Song XQ Pei L Wang XM Comparative study of the replication of infectious bursal disease virus in DF-1 cell line and chicken embryo fibroblasts evaluated by a new real-time RT-PCR J Virol Methods 2009 157 205 210 10.1016/j.jviromet.2009.01.001 19186190

[B15] Blomstrom AL Hakhverdyan M Reid SM Dukes JP King DP Belak S Berg M A one-step reverse transcriptase loop-mediated isothermal amplification assay for simple and rapid detection of swine vesicular disease virus J Virol Methods 2008 147 188 193 10.1016/j.jviromet.2007.08.023 17920701

[B16] Endo S Komori T Ricci G Sano A Yokoyama K Ohori A Kamei K Franco M Miyaji M Nishimura K Detection of gp43 of *Paracoccidioides brasiliensis *by the loop-mediated isothermal amplification (LAMP) method FEMS Microbiol Lett 2004 234 93 97 10.1111/j.1574-6968.2004.tb09518.x 15109725

[B17] Mao XL Zhou S Xu D Gong J Cui HC Qin QW Rapid and sensitive detection of Singapore grouper iridovirus by loop-mediated isothermal amplification J Appl Microbiol 2008 105 389 397 10.1111/j.1365-2672.2008.03761.x 18312563

[B18] Nagamine K Watanabe K Ohtsuka K Hase T Notomi T Loop-mediated isothermal amplification reaction using a nondenatured template Clin Chem 2001 47 1742 1743 11514425

[B19] Yoneyama T Kiyohara T Shimasaki N Kobayashi G Ota Y Notomi T Totsuka A Wakita T Rapid and real-time detection of hepatitis A virus by reverse transcription loop-mediated isothermal amplification assay J Virol Methods 2007 145 162 168 10.1016/j.jviromet.2007.05.023 17604128

[B20] Zhang QL Shi CY Huang J Jia KT Chen XH Liu H Rapid diagnosis of turbot reddish body iridovirus in turbot using the loop-mediated isothermal amplification method J Virol Methods 2009 158 18 23 10.1016/j.jviromet.2009.01.008 19187785

[B21] Cho HS Park NY Detection of canine distemper virus in blood samples by reverse transcription loop-mediated isothermal amplification J Vet Med B Infect Dis Vet Public Health 2005 52 410 413 16283921 10.1111/j.1439-0450.2005.00886.x PMC7165947

[B22] Sun ZF Hu CQ Ren CH Shen Q Sensitive and rapid detection of infectious hypodermal and hematopoietic necrosis virus (IHHNV) in shrimps by loop-mediated isothermal amplification J Virol Methods 2006 131 41 46 10.1016/j.jviromet.2005.07.011 16214229

[B23] Higashimoto Y Ihira M Ohta A Inoue S Usui C Asano Y Yoshikawa T Discriminating between Varicella-Zoster virus vaccine and wild-type strains by loop-mediated isothermal amplification J Clin Microbiol 2008 46 2665 2670 10.1128/JCM.00216-08 18550736 PMC2519472

[B24] Chettle N Stuart JC Wyeth PJ Outbreak of virulent infectious bursal disease in East Anglia Vet Rec 1989 125 271 272 10.1136/vr.125.10.271 2552640

[B25] Nunoya T Otaki Y Tajima M Hiraga M Saito T Occurrence of acute bursal disease with high mortality in Japan and pathogenicity of field isolates in specific pathogen free chicken Avian Dis 1992 36 597 609 10.2307/1591754 1329709

[B26] Lombardo E Maraver A Espinosa I Fernandez-Arias A Rodriguez JF VP5, the nonstructural polypeptide of infectious bursal disease virus, accumulates within the host plasma membrane and induces cell lysis Virology 2000 277 2 345 357 10.1006/viro.2000.0595 11080482

[B27] Yao K Goodwin MA Vakharia VN Generation of a mutant infectious bursal disease virus that does not cause bursal lesions J Virol 1998 72 4 2647 2654 9525581 10.1128/jvi.72.4.2647-2654.1998 PMC109706

